# Camera-Based System for Drafting Detection While Cycling

**DOI:** 10.3390/s20051241

**Published:** 2020-02-25

**Authors:** Gianni Allebosch, Simon Van den Bossche, Peter Veelaert, Wilfried Philips

**Affiliations:** 1 TELIN-IPI, Ghent University, Sint-Pietersnieuwstraat 41, Gent B-9000, Belgium; sidnboss.VandenBossche@ugent.be; 2 imec, Kapeldreef 75, Leuven B-3001, Belgium

**Keywords:** computer vision, triathlon, cycling, object detection, object tracking, distance determination, probability theory

## Abstract

Drafting involves cycling so close behind another person that wind resistance is significantly reduced, which is illegal during most long distance and several short distance triathlon and duathlon events. In this paper, a proof of concept for a drafting detection system based on computer vision is proposed. After detecting and tracking a bicycle through the various scenes, the distance to this object is estimated through computational geometry. The probability of drafting is then determined through statistical analysis of subsequent measurements over an extended period of time. These algorithms are tested using a static recording and a recording that simulates a race situation with ground truth distances obtained from a Light Detection And Ranging (LiDAR) system. The most accurate developed distance estimation method yields an average error of 0.46 m in our test scenario. When sampling the distances at periods of 1 or 2 s, simulations demonstrate that a drafting violation is detected quickly for cyclists riding at 2 m or more below the limit, while generally avoiding false positives during the race-like test set-up and five hour race simulations.

## 1. Introduction

As in most endurance sports, the goal of triathlon races is to reach the finish line as quickly as possible. In addition to optimizing training, rest, and nutrition, triathletes can greatly improve their performance by working on a streamlined posture. A huge advantage can also be gained by cycling closely behind another competitor during a race. This other competitor then acts as a wind shield. This technique is called drafting, stayering, or slipstreaming. Recent studies suggest using this technique by riding in a peloton can reduce aerodynamic drag by 90–95% compared to that of an isolated cyclist [1].

The International Triathlon Union (ITU) makes a distinction between draft-legal and draft-illegal races [2]. Most long or middle distance races and many standard or sprint distance races fall under the latter category, where it is forbidden to draft behind another athlete or a motor vehicle, i.e., to enter the bicycle or vehicle drafting zone for an extended period of time. For standard and shorter distances the bicycle draft zone is 10 m long measured from the leading edge of the front wheel. An athlete is allowed to enter another athlete’s draft zone but must be progressing through that zone. A maximum of 20 s is allowed to pass through another athlete’s drafting zone. The official regulations regarding middle and long distance events are slightly different, enforcing 12 m distance and 25 s maximal duration. Note that some race organizers enforce even other distances or durations. The experiments in this paper, always assume a 10 m drafting zone and 20 s maximal duration, unless stated otherwise.

**Figure 1:**
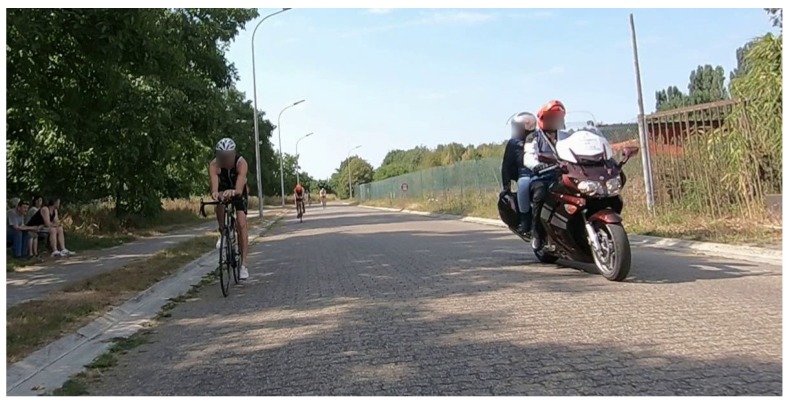
Typical drafting rule check in a triathlon race. A motorbike rides next to the athletes, and the referee performs a visual estimate of the distance between them. If the estimated distance is too small (e.g., smaller than 10 m) for an extended period (e.g., longer than 20 s), the drafting athlete receives a penalty.

Today, drafting regulations are monitored by referees driving a motorbike (Figure 1). However, monitoring these rules in practice is difficult, hence unpunished drafting violations do occur frequently. In a typical triathlon race, there are not enough referees to check all triathletes all the time. In addition, the referees’ decisions are still subjective as they can only roughly estimate the distances between the bicycles. Furthermore, athletes can typically hear a motorbike approaching them, so they can adjust their behavior momentarily.

The lack of reliable detection of illegal drafting can lead to arbitrariness in the best case, and possibly even to corruption, such as favoring some individuals in the worst case. A drafting detection system based on video taken from a camera mounted under the saddle of a bicycle, equipped with computer vision techniques can offer a solution to these problems. Moreover, when an athlete challenges a referee’s decision, a video based system and its recorded distances can provide supportive data. In summary, by continuously checking compliance with the drafting rules, a camera based detection system will lead to less drafting and consequently ensure fairer triathlon or duathlon races.

In a previous implementation of a drafting detection system, GPS and Web Services are used, yielding an average accuracy of 1.32 m for the absolute position of each athlete [3]. The error can increase in cases of urban canyons and tree lines, because of the limitations of the GPS device, which is a significant deviation, given the drafting distance limit. Two more accessible solutions for a drafting detection system are: Light Detection And Ranging (LiDAR) and RAdio Detection And Ranging (RADAR). The former is a very accurate but expensive and non-compact technique. The latter is more compact and cheaper. However, for object detection with a RADAR system, a sufficient number of reflection points are required at known positions, making this solution potentially less reliable.

Monocular vision based detection and distance estimation algorithms have been demonstrated to successfully estimate the distance between cars in literature for traffic applications. These techniques typically combine object detection networks with geometric techniques but also employ extra information to accurately estimate the distances. These features include the presence of lane markings [4], or the specific geometry of cars [5]. However, in the application of detecting bicycles in triathlon races, neither of these additional features can be used.

In this paper, we present a video based solution that automates the task of assessing potential drafting violations and extend the work of Van den Bossche et al. [6]. A single camera which looks backwards is mounted under the saddle of the bicycle. We do not consider a stereo camera set-up with two or more cameras on one bicycle because this would mitigate the benefit of compactness, and add to the cost.

To be used in a triathlon race context, the mechanical robustness of the system is very important, because road conditions may often cause problems. It must be able to deal with vibrations in the image due to rough road surfaces, such as cobble stones or speed bumps. Furthermore, installing the system on a race bike should be a simple and straightforward procedure. In the proposed system, the mounting of the camera only involves the camera height and the tilt angle, which are easy to control.

The focal point of this paper is the detection of drafting by the camera, whose operation can be summarized as follows. Bicycles are detected in the individual video images with a Convolutional Neural Network (CNN) architecture. The bicycle closest behind the camera is tracked and the distance to the bicycle with the camera is estimated from the apparent height and position of the bicycle. The distance is monitored over an extended time period, which enables an estimate of the probability that a drafting violation (e.g., longer than 20 s in the draft zone) has occurred.

The main contributions of this paper can be summarized as follows:

1. We trained, applied, and analyzed the performance of real-time CNN-based object detector, specifically for detecting race, time trial, and triathlon bicycles.
2. We describe two methods for estimating the distance from the camera to the cyclists behind. We performed sensitivity analysis and investigated the systematic errors that can occur which are caused by making simplifying assumptions. The accuracy of the distance estimators is also verified in a realistic scenario using a Light Detection And Ranging (LiDAR) scanner.
3. We developed an efficient method which determines the probability of violating the drafting rule, based on successive distance estimations and a model of the measurement error. The behavior of this method is rigorously tested in a realistic scenario and through the use of simulations.

## 2. Drafting Detection

In this section, we discuss all processing steps in our proposed approach. Note that (as a preprocessing step), we assume the camera is intrinsically calibrated and the lens distortion was removed, e.g., with the method of Zhang [7].

### 2.1. Bicycle Detection

**Figure 2:**
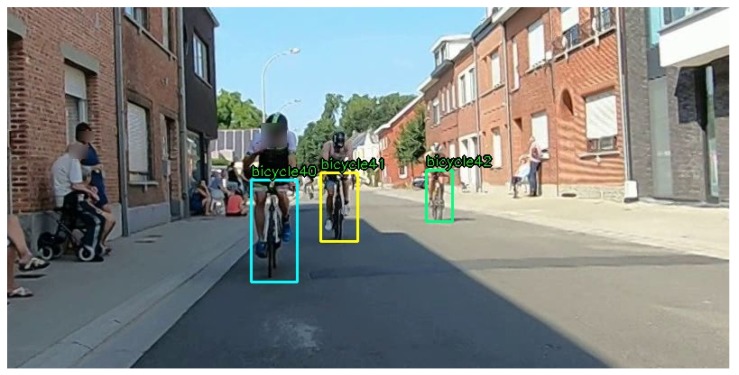
Example of a frame with manually annotated bounding boxes from our training set, captured at a non-drafting triathlon race in Kapelle-op-den-Bos, Belgium.

The first step in the drafting detection system is to detect cyclists in real-time. The aim is to have a robust, real-time detection at least up to 20 m from the camera. To achieve this, a Tiny YOLOv3 (You Only Look Once, version 3) [8] network has been trained for specifically detecting triathlon bikes, using 4 different triathlon race recordings of about one hour long each. Our training set for the object detector is very diverse, also including lighting changes (e.g., in tunnels) and poor weather conditions, such as rain. The detector was trained with approximately 60,000 manually annotated ground truth bounding boxes, which overlay the bicycle from the ground (bottom of the front wheel) to the handlebars. An example frame with manually annotated boxes is shown in Figure 2. This renders the size and position of the bounding boxes useful for distance estimation, as will be shown in Section 2.3. The training versus test set ratio was 80 to 20%.

The tiny YOLOv3 network obtained an Average Precision (AP) of 77.19% on our dataset, by comparing the detections with manually annotated ground truth bounding boxes. On our hardware (1080p input video and desktop PC with a GeForce GTX 1060 GPU), the detection speed on the test set is 42.57 fps. We note that other object detectors, such as YOLOv3 [8], Faster RCNN (Region Convolutional Neural Network) [9], and Mask RCNN [10], also have the potential to yield high detection rates but are slower, taking 24.58, 7.85, and 6.64 fps, respectively, on the same hardware set-up.

The Single Shot multibox Detector (SSD) [11] does provide an interesting alternative. It is based on the MobileNet CNN architecture, was specifically designed for constrained devices (e.g., smart phones), and yields a similar processing speed as Tiny YOLOv3 (42.25 fps). Depending on the final application, and whether the processing needs to be executed on the device (i.e., attached to the bicycle) or off-line after the race, a more heavyweight but also more accurate and slower network architecture could be used.

The performance of a re-trained version of this network remains to be investigated in future work. Note that the AP of the detector is further improved by applying object tracking on the detected bicycles, which is discussed in the following subsection.

### 2.2. Bicycle Tracking

Because the regulations also incorporate a maximal drafting duration, the detected bicycles need to be tracked as long as they are visible throughout the video. We are only interested in tracking a bicycle as long as it stays (visible) behind the camera bicycle. In addition, note that, in order to be robustly detected by Tiny YOLOv3 and tracked, the bicycle should be reasonable close. In our experiments, Tiny YOLOv3 was able to accurately detect bicycles which were further than 20 m away, which is still well above the typical drafting limit.

In our method, the bounding box of the bicycle closest to the camera is not only detected but also tracked and its trajectory is recorded. This results in reliable samples to estimate the distance, which will be discussed in the next section. We note that other potential cyclists (riding further from the camera) are discarded in our current method. To be used in a realistic application, the system should be able to detect multiple cyclists behind. However, due to the increased complexity of the track management of such a system, we have not yet investigated this in our current proposed method.

Tracking increases the robustness of the draft detection considerably. The detection miss rate is lowered by using the predicted position of the tracker in case the detector misses a detection for a certain frame. If a bicycle is detected in the previous frame but not in the current frame, its current position can still be estimated from a tracker’s prediction step. Thus, the effect of missed detections (False Negatives) is mitigated and bicycles in the scene can be better monitored continuously. More specifically, bicycles are linked when they overlap in successive frames. This strategy is simple but accurate (as will be demonstrated further in this subsection), because of the high frame rate and low relative speeds. When no overlapping bounding box is detected at a given point in time, the tracker uses the last detected bounding box as input and then tries to locate it in the current frame. When a new bounding box is detected, a new track is initialized. In this way, the same bicycle can be uniquely identified by its ‘bicycle id’.

**Figure 3:**
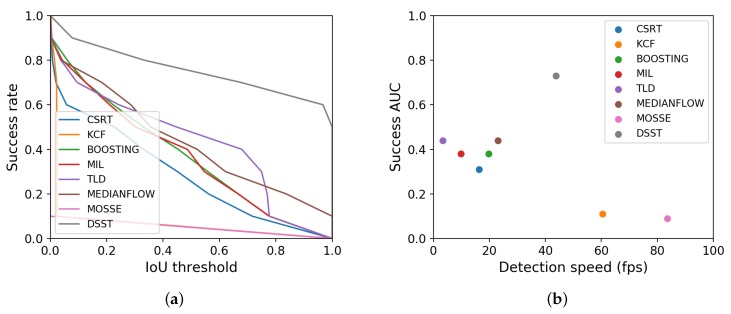
Tracking benchmark for CSRT (Channel and Spatial Reliability Tracker) [12], KCF (Kernelized Correlation Filters) [13], Boosting [14], MIL (Multiple Instance Learning) [15], TLD (Tracking, Learning, and Detection) [16], Medianflow [17], MOSSE (Minimum Output Sum of Squared Error) [18], and DSST (Discriminative Scale Space Tracking) [19]. (**a**) Success plot: success rate versus Intersection Over Union (IoU) threshold. (**b**) Area Under Curve (AUC) of (**a**) versus execution speed.

In order to make an informed decision with regard to the object tracker, eight object trackers from the literature are benchmarked on our dataset. The success AUC is defined as the Area Under the Curve (AUC) of the success plot, which demonstrates the percentage of the number of frames where the Intersection Over Union (IOU) of the estimated bounding box and the ground truth bounding box is larger than the considered threshold. We evaluated state-of-the-art trackers according to detection speed and success rate as shown in Figure 3: CSRT (Channel and Spatial Reliability Tracker) [12], KCF (Kernelized Correlation Filters, Copyright (c) 2012, Piotr Dollar All rights reserved; Copyright (c) 2014, Tomáš Vojíř.) [13], Boosting [14], MIL (Multiple Instance Learning) [15], TLD (Tracking, Learning, and Detection) [16], Medianflow [17], MOSSE (Minimum Output Sum of Squared Error) [18], and DSST (Discriminative Scale Space Tracking) [19].

According to this benchmark, the Discriminative Scale Space Tracking (DSST) object tracker is the most appropriate solution. It clearly stands out with respect to the AUC of the success plot (0.73 versus 0.44 for the second highest scoring method) and has the third highest processing speed (still well above real time). The DSST tracker builds on the MOSSE tracker (described in Bolme et al. [18]) and extends it with a multi-scale pyramid to estimate the scale of an object. Note that rotation invariance is less important in our application, since the orientation of the cyclist only typically changes in a road bend.

**Figure 4:**
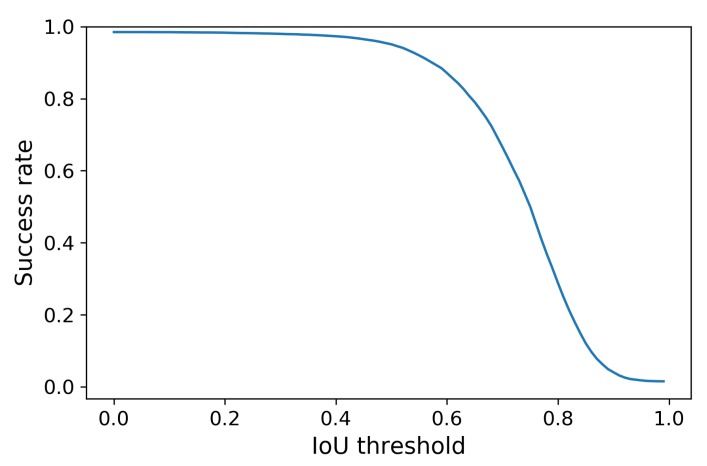
Success plot for the combination of Tiny YOLOv3 (You Only Look Once, version 3) + DSST.

The Tiny YOLOv3 object detector is tested in combination with the DSST object tracker with regard to improvement in the detection rate. An initial AP of 77.19% for Tiny YOLOv3 was obtained, but, after applying object tracking, an increase of the recall yields an average precision of 88.27% for the bounding boxes. The success plot of this combination is shown in Figure 4.

Note that this combination is notably very reliable when the IoU threshold is allowed to be low. However, in some cases, a higher IoU threshold is preferred, e.g., if the height of the bounding box needs to be very accurate. Figure 3 suggests that relying more on the DSST tracker (as opposed to using it mainly for missed detections) could increase the combined detection/tracking performance further. Other configurations could be investigated in future work.

### 2.3. Distance Estimation

**Figure 5:**
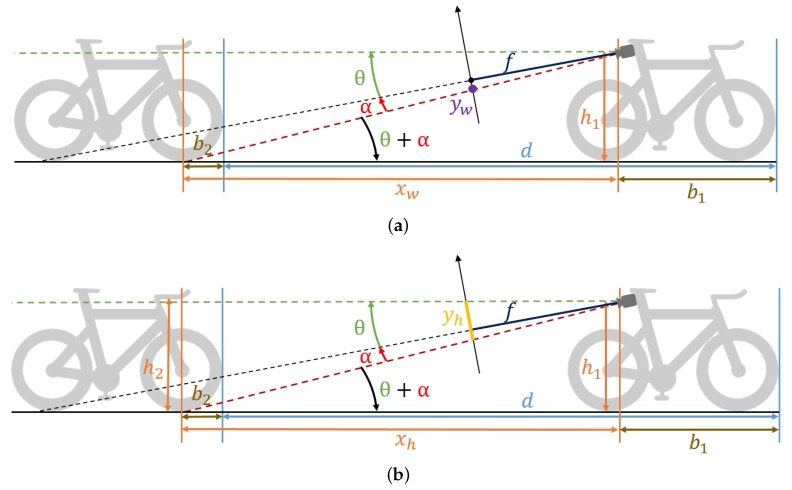
Demonstration of how the distance can be computed from the position of the bounding box. The distance *d* between the two athletes is measured between the leading edges of their bicycles’ front wheels. In the proposed methods, we estimate the distance $x=x_{w}=x_{h}$, which differs from *d* by a fixed constant distance $\left(b_{1}-b_{2}\right)$. This figure was modified from References [20,21]. (**a**) Wheel Position-Based method (WPm), estimated from $y_{w}$; (**b**) Handlebar Height-Based method (HHm), estimated from $y_{h}$.

In this section, we discuss two alternatives to estimate the distance between the camera bicycle and the bicycle behind: the Wheel Position-Based method (WPm) and the Handlebar Height-Based method (HHm). Each method has a benefit with respect to the other, which is discussed in detail further in this section. For both methods, we assume a flat road and known geometry of the bicycles, as shown in Figure 5. At the end of the section, we perform a sensitivity analysis w.r.t. potential violations of the assumptions made and describe systematic errors related to these assumptions.

In both methods, we derive *x* from the position of the bounding box (with bottom at $y_{w}$ and height $y_{h}$) of a detected cyclist. The camera tilt angle θ, the focal length *f* and the camera height $h_{1}$ have known values. The camera is mounted at the rear of the saddle and, according to the rules the drafting distance, *d* is calculated from front wheel to front wheel. Thus, we must add $\left(b_{1}-b_{2}\right)$ to *x* to obtain the distance between the two front wheels.

To distinguish between the two models, we will denote the distances estimated by the two methods as $x_{h}$ and $x_{w}$, respectively. In the ideal case, we should have $x_{h}=x_{w}=x$.

#### 2.3.1. Wheel Position-Based Method (WPm)

The first method estimates the location of the bottom of the wheel on the ground plane, starting from a known tilt angle θ and camera height $h_{1}$ (Figure 5a). Note that the WPm is closely related to the camera-pose-based trigonometric vehicle distance estimation method described in Reference [22]. Let α denote the angle between the bottom of the bounding box and the camera center line; thus,

(1) $$\alpha =\arctan\frac{-y_{w}}{f}\;,$$

where $y_{w}$ is the vertical distance from the image center to the bottom of the bounding box in the image, and *f* is the focal length of the camera. The distance $x_{w}$ thus equals

(2) $$\begin{array}{cc} x_{w} & =\frac{h_{1}}{\tan(\alpha +\theta )} \end{array}$$

(3) $$\begin{array}{cc} \; & =\frac{h_{1}}{\tan\left(\arctan\frac{-y_{w}}{f}+\theta \right)} \end{array}$$

(4) $$\begin{array}{cc} \; & =\frac{h_{1}\left(f+y_{w}\tan\theta \right)}{-y_{w}+f\tan\theta }\;. \end{array}$$

The advantage of this method is that is independent of the height of the bicycle behind, which typically can only be estimated. A possible disadvantage of this method is the strong dependence of Equation (4) on the tilt angle of the camera, which will be demonstrated in the sensitivity analysis in Section 2.3.3.

#### 2.3.2. Handlebar Height-Based Method (HHm)

The second distance estimation method is based on the height of the handlebars above the ground, which is obtained from the height of the detected and tracked bounding box. The expression for the distance of to the object w.r.t. the height of the bounding box is complex and depends on more parameters than the WPm (see Appendix A). However, this expression can be simplified if we assume that $h_{1}\approx h_{2}$. From Equation (4),

(5) $$x_{h}=\frac{h_{1}\left(f\left(1+\tan^{2}\theta \right)-y_{h}\tan\theta \right)}{y_{h}}\;,$$

where $y_{h}=f\tan\theta -y_{w}$ is the height of the bounding box. For realistic recordings a small tilt angle is expected and $y_{h}\ll f$ when the detected object is far enough from the camera. From Figure 5, it can be deduced that $y_{h}=f\left(\tan\theta +\tan\alpha \right)=f\tan\left(\theta +\alpha \right)\left(1-\tan\theta \tan\alpha \right)=\frac{fh}{x}\left(1-\tan\theta \tan\alpha \right)$. The vertical FOV of commercial cameras is rarely larger than 90°; thus, |tanα|≤1 when the detected object is entirely visible. Hence, for a small tilt angle θ, $y_{h}\approx \frac{fh}{x}$. In a typical set-up, *h* is more than ten times smaller than the drafting distance limit, so $y_{h}\ll f$ for the most crucial situations. Thus,

(6) $$x_{h}\approx \frac{fh_{1}}{y_{h}}=x_{h}{|}_{\theta =0}\;.$$

**Figure 6:**
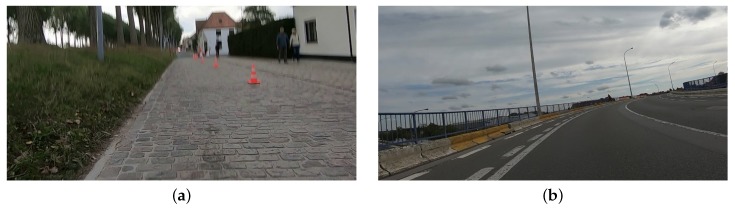
Examples of situations where the road surface is not flat: (**a**) cobbled road; (**b**) bridge deck.

The fact that the tilt angle θ can be ignored is a significant advantage of this method. The initial tilt angle must not be measured when setting up the system. Furthermore, due to vibrations, the camera position might change during the recording. Finally, there are situations where the road itself is not perfectly flat, as shown in Figure 6, which influences the distance estimation in a similar fashion to **that of camera rotation. An in-depth sensitivity analysis is performed in the next section**.

#### 2.3.3. Sensitivity Analysis

The parameters in the distance calculation formulae Equations (4)–(6) either depend on the scene geometry ($h_{1}$ and θ), the intrinsic camera properties (*f*) or the position or height of the detected and tracked object in the image ($y_{w}$ and $y_{h}$). The bicycle’s speed is not included in the distance or probability calculations, so it does not directly contribute the error. However, it might contribute indirectly, e.g., by causing more jitter on an uneven road surface, or when the relative speed between the two bicycles is high, which might (albeit slightly) influence the performance of our tracker.

In practice, all parameters can be prone to errors, each having different potential causes. Hence, we performed sensitivity analysis of $x_{h}$ and $x_{w}$ w.r.t. these parameters, which is discussed below. The full analysis is performed in Appendix B. An overview of the results for a small tilt angle θ is demonstrated in Table 1. For example, when there is a small error in the measurements of the height $h_{1}$, the relative error on the distance calculated by the WPm satisfies $\Delta x_{w}\;/\;x_{w}\approx \Delta h_{1}\;/\;h_{1}$.

The sensitivity analysis demonstrates that measured errors w.r.t. camera height $h_{1}$, the focal length *f*, the bottom position bounding box $y_{w}$ or the height of the bounding box $y_{h}$ are all (approximately) proportionally propagated to the estimated distances $x_{h}$ and $x_{w}$, i.e., an error of 5% on one of these parameters yields a 5% error on the estimated distance. This also indicates that the absolute error increases linearly with the distance to the cyclist behind.

The partial derivative for $x_{h}$, estimated by the HHm, w.r.t. θ is relatively insensitive to tilt angle estimation errors when α (see Figure 5) is small. This justifies the relaxation from Equation (5) to Equation (6). For the WPm, however, even a small estimation error for θ can potentially lead to a significant error in the estimation of $x_{w}$, notably when α is small.

**Figure 7:**
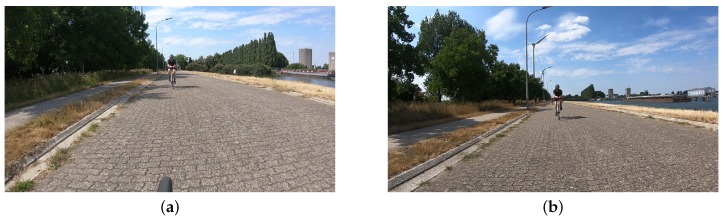
Example of an unwanted, significant change in tilt angle for one sequence, likely after hitting a rough road patch; (**a**) back wheel still visible; (**b**) back wheel no longer visible.

If the camera cannot be very tightly fixed in its original position, the tilt angle is the most error-prone parameter. In this scenario, this angle can change (undesirably) throughout a recording, e.g., due to vibrations as demonstrated in Figure 7. Thus, when using the WPm, this parameter needs to be updated at runtime. This can be realized by optical flow analysis or by utilizing information from an accelerometer and/or a gyroscope.

#### 2.3.4. Systematic Errors

The proposed HHm makes two assumptions; $h_{1}\approx h_{2}$ and θ≈0°. In this section, the effect of the systematic error introduced by these simplifications is investigated.

In practice, there can be a height difference between $h_{1}$ and $h_{2}$ of the order of a few centimeters. This issue could be resolved, e.g., by adding a distinctive marker on all bicycles at a predetermined height, which can be detected by the software, and internally adjusting the value of $h_{2}$ in the detection step accordingly.

The tilt angle θ typically differs from 0° when the camera position is either redirected during set-up, or when the angle changes due to vibrations (Figure 7) during the bike ride. A typical tilted camera has a magnitude of θ of up to 10°.

**Figure 8:**
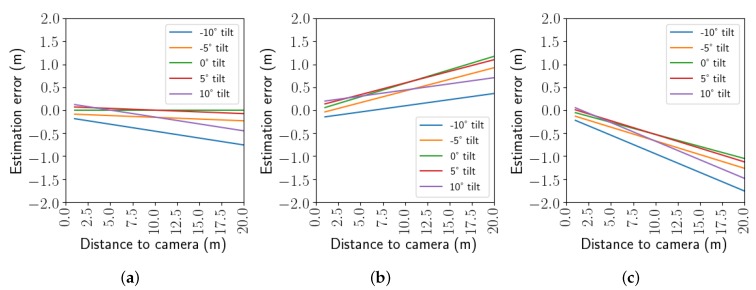
Systematic errors made by the HHm by simplifying the full equation Equation (A3) to Equation (6) in different camera set-ups. The focal length was set to 1000 pixels. (**a**) $h_{1}=h_{2}=0.9$ m, (**b**) $h_{1}=0.9$ m, $h_{2}=0.85$ m, (**c**) $h_{1}=0.9$ m, $h_{2}=0.95$ m.

The difference between the exact solution (see Appendix A, Equation (A3)) and the approximated solution in Equation (6) for $x_{h}$ is analyzed and demonstrated in Figure 8 for different realistic values of θ, $h_{1}$ and $h_{2}$.

The systematic errors vary linearly w.r.t. the distance from the camera. A camera tilt (|θ|>0°) or $h_{1}<h_{2}$ contributes to an underestimation of the estimated distance, notably for objects further away from the camera. When $h_{1}>h_{2}$, the true distance is similarly overestimated.

The WPm is not dependent on $h_{2}$, so it is expected this method performs better when the height of the handlebar of the cyclist behind cannot be accurately estimated. In Section 3, the performance of both methods is evaluated through simulations and in a realistic scenario.

### 2.4. Drafting Probability

In the previous sections, methods for determining the distance between two bicycles at a given point in time were explored, based on object detection and tracking. Since the drafting rule also has a temporal component, it is necessary to robustly combine multiple measurements of the distance over a given time period. Given a set of measurements and a certain measurement error probability density function (pdf), our end result is an estimated probability that the cyclist behind violated the drafting rule.

#### 2.4.1. Theoretical Probability Determination

The probability of drafting for a given period of time is closely related to the known theory of ‘success runs’ [23]. However, the main difference in this application is that the probability of ‘success’ depends on the measured distance, which changes between measurements.

Let $q_{1:n}$ be a binary vector of length *n*. When the distance between the two bicycles *d* is smaller than the drafting distance limit $d_{L}$ (e.g., 10 m) at *t*, $q_{t}=1$; otherwise, $q_{t}=0$.

We assume that the likelihood function of the real distance *d* between two bicycles can be modeled as a distance-dependent normal distribution, with standard deviation $\sigma_{x}$ at $x_{t}$. We assume here that the measured distance $x_{t}+\left(b_{1}-b_{2}\right)$ is close to the real distance *d*. Thus, $\sigma_{x}=\sigma \left(x_{t}+\left(b_{1}-b_{2}\right)\right)\approx \sigma \left(d\left(t\right)\right)$. Let $x_{t}$ be a distance measurement at time instance *t*, with fixed offset $b_{1}-b_{2}$ (see Figure 5). The probability that the bicycle behind is actually closer than $d_{L}$ at one time instance can thus be expressed as

(7) $$P\left(q_{t}=1|x_{t}\right)=P\left(d\left(t\right)<d_{L}|x_{t}\right)=\frac{1-\mathrm{erf}\left(\frac{-d_{L}+\left(x_{t}+\left(b_{1}-b_{2}\right)\right)}{\sqrt{2}\sigma_{x}}\right)}{2}\;.$$

Similarly, the probability that the bicycle is not closer than $d_{L}$ is $P\left(q_{t}=0|x_{t}\right)=1-P\left(q_{t}=1|x_{t}\right)$.

To compute the probability of $q_{1:n}$ given a set of measurements $x_{1:n}$, we define the index sets $I_{1}=\left\{t\in \left[1,n\right]|q_{t}=1\right\}$ and $I_{0}=\left\{t\in \left[1,n\right]|q_{t}=0\right\}$. We also assume that all measurements are independent and that the probability that q occurs, given successive measurements $x_{1:n}=\left(x_{1},x_{2}\ldots x_{n}\right)$, can be computed as

(8) $$P\left(q_{1:n}|x_{1:n}\right)=\prod_{t\in I_{1}}P\left(q_{t}=1|x_{t}\right)\prod_{t\in I_{0}}P\left(q_{t}=0|x_{t}\right).$$

$q_{1:n}$ is defined as a ‘valid drafting pattern’ ($q_{1:n}\in Q_{v}$) if it contains at least one instance of at least $k=\lceil T_{L}\;/\;f_{s}+1\rceil$ successive samples with value 1, where $f_{s}$ is the sampling rate and $T_{L}$ the maximal time an athlete is allowed to stay in the drafting zone (e.g., 20 s). Since all patterns are mutually exclusive, the probability of drafting over a given period of time (*n* samples) is thus the sum of the probabilities of the occurrence of all valid drafting patterns. Let $v_{1:n}$ be the event of a drafting rule violation for t∈[1,n]. The probability that such an event has occurred, given distance measurements $x_{1:n}$ is

(9) $$P\left(v_{1:n}|x_{1:n}\right)=\sum_{q_{1:n}\in Q_{v}}P\left(q_{1:n}|x_{1:n}\right)\;.$$

Note that $P(v_{1:n})$ always increases over time, i.e., if n<m, then $P\left(v_{1:n}|x_{1:n}\right)\leq P\left(v_{1:m}|x_{1:n}\right)$. Since $0\leq P(q_{t}=1|x_{t})\leq 1$ and $P\left(q_{t}=1|x_{t}\right)=1-P\left(q_{t}=0|x_{t}\right)$, it is easy to show that

(10) $$\sum_{q_{1:n}\in {\left\{0,1\right\}}^{n}}P\left(q_{1:n}|x_{1:n}\right)=1\;.$$

Consequently, since $Q_{v}\subseteq {\left\{0,1\right\}}^{n}$,

(11) $$0\leq \sum_{q_{1:n}\in Q_{v}}P\left(q_{1:n}|x_{1:n}\right)\leq 1\;.$$

In our application, the drafting violation probability should approach 1 as soon as the cyclist behind spends longer than the drafting time limit $T_{L}$ in the drafting zone. On the other hand, when a cyclist stays outside of the drafting zone, or is only inside for less than $T_{L}$, the probability should stay close to 0. Three factors determine how well $P(v_{1:n}|x_{1:n})$ follows these considerations:

1. Systematic errors: when one or more parameters in the distance estimation formulae Equations (4)–(6) are erroneously set, this leads to a systematic over- or underestimation of the distance.
2. The distance estimation noise: less noise means more certainty about the estimated distance; thus, the individual probabilities in Equation (7) are closer to either 0 or 1. In Section 3.2, we will investigate the noise level for our test set-up and through simulations.
3. The sampling rate: for independent measurements and a given noise level and distance, there is always a higher probability that at least one of the measurements is far off from the real distance, which can significantly influence the calculated probability in Equation (9). Conversely, when the sampling rate is low, there is a higher chance of sampling bias. Hence, a trade-off exists, which will be investigated in Section 3.2.

#### 2.4.2. Efficient Probability Calculation

A naive method of calculating the drafting probability would consist of an exhaustive summation of all probabilities for any given pattern (time complexity $O(2^{n})$), which in turn require *n* multiplications of individual likelihoods, each calculated from Equation (7).

However, it is also possible to calculate the drafting probability with time complexity O(n) and constant space complexity by re-using previous results. Assume that all earlier probabilities of drafting $P(v_{1:n-i}|x_{1:n})$ for t∈[1,n−1] are known. The updated probability of drafting is now the sum of the probability that drafting had already occurred and the probability that it is the first time the drafting limit has been exceeded for longer than *k* successive samples. The drafting limit can only be exceeded for the first time if the last *k* samples of $q_{1:n}$ (i.e., $q_{n-k+1:n}$) are 1, the one before that is equal to 0 and no other valid drafting patterns can be found in $q_{1:n-k-1}$. Hence, the probabilities can be calculated by the following recursive expression:

(12) $$\begin{array}{c} P\left(v_{1:n}|x_{1:n}\right)=P\left(v_{1:n-1}|x_{1:n-1}\right)+\left(1-P\left(v_{1:n-k-1}|x_{1:n-l-1}\right)\right)\left(P\left(q_{n-k}=0\right)|x_{n-k}\right)P\left(v_{n-k+1:n}|x_{n-k+1:n}\right), \end{array}$$

(13) $$\begin{array}{c} =P\left(v_{1:n-1}|x_{1:n-1}\right)+\left(1-P\left(v_{1:n-k-1}|x_{1:n-k-1}\right)\right)\left(P\left(q_{n-k}=0|x_{n-k}\right)\right)\prod_{t=n-k+1}^{n}P\left(q_{t}=1|x_{t}\right)\;. \end{array}$$

This equation demonstrates that only the last *k* measured distances (or probabilities of momentary drafting with Equation (7)) and the last k+1 calculated drafting rule violation probabilities need to be kept in memory and a constant number of calculations is done for every new sample.

## 3. Evaluation and Results

In the previous section, we proposed two strategies to estimate the distance between two bicycles: the HHm and the WPm. Both methods are analyzed and benchmarked in order to make a well-founded choice concerning robustness and accuracy. To achieve this, two types of test recordings have been made and are discussed: a static and a dynamic situation test. Both tests were executed in sunny weather conditions. The probability of drafting that is inferred from these measurements is evaluated in the final part of this section.

### 3.1. Distance Estimation

#### 3.1.1. Static Test

**Figure 9:**
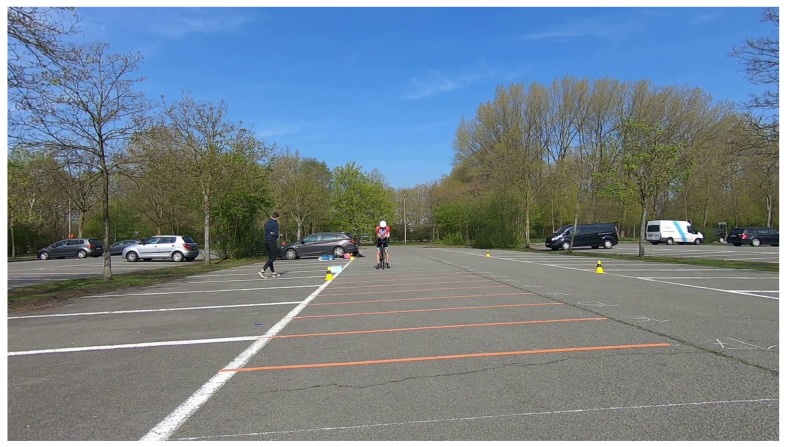
Example of static distance calculation test. The camera bicycle is placed at the 0 m mark, and the cyclist behind takes place at one of the other marks, each spaced 1 m apart.

To determine the accuracy of the calculated distance, a static situation for well-known reference distances is considered as shown in Figure 9. The bicycle with the camera is fixed at the 0 m mark. Another bicycle is placed at distances ranging from 1 m to 20 m to the camera, at regular intervals of 1 m each. For this test, an average error of the measured distances over 100 frames is plotted for each ground truth distance.

**Figure 10:**
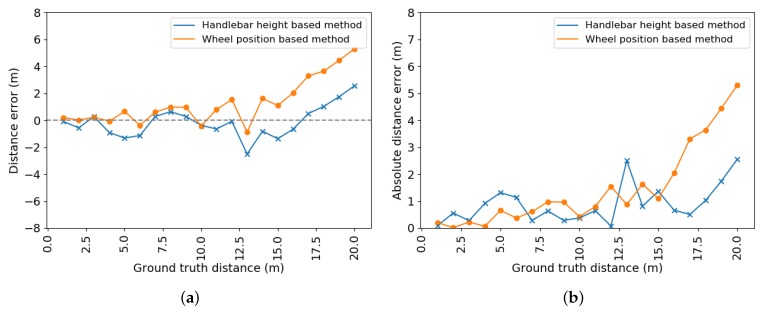
Error plots for the static test for both distance estimation methods. (**a**) Signed error; (**b**) absolute error.

The results of the HHm and the WPm are shown in Figure 10. In this scenario, the WPm obtains better overall results for distances less than or equal to 6 m. For larger distances, the HHm produces a lower overall absolute distance error. Note that these measurements have been realized with a known tilt angle; therefore, the tilt angle deviation is assumed to be close to zero for the wheel-based method.

The limitation of this static situation test is that it is considerably different from what happens during a triathlon race, since only one, static athlete is visible at any given time. Moreover, the athlete supports the bicycle by standing on the ground with one or two legs, which provides a slightly different context for the trained object detector and might thus lead to inferior results. Hence, a dynamic situation test is discussed in the following paragraph.

#### 3.1.2. Dynamic Test

**Figure 11:**
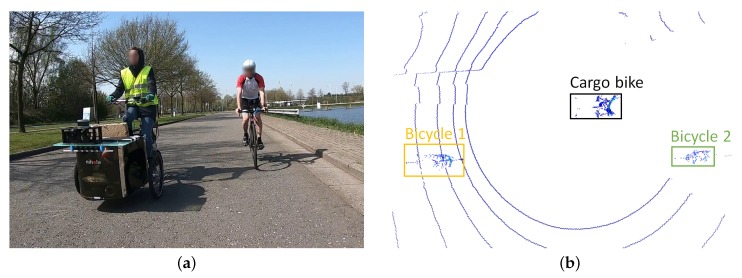
Example of dynamic distance calculation test. An electric cargo bicycle with mounted Light Detection And Ranging (LiDAR) scanner drives next to the cyclists. The LiDAR data provides ground truth for the distance determination. (**a**) Input image from the camera mounted on Bicycle 1; (**b**) top view of the LiDAR scanlines.

**Figure 12:**
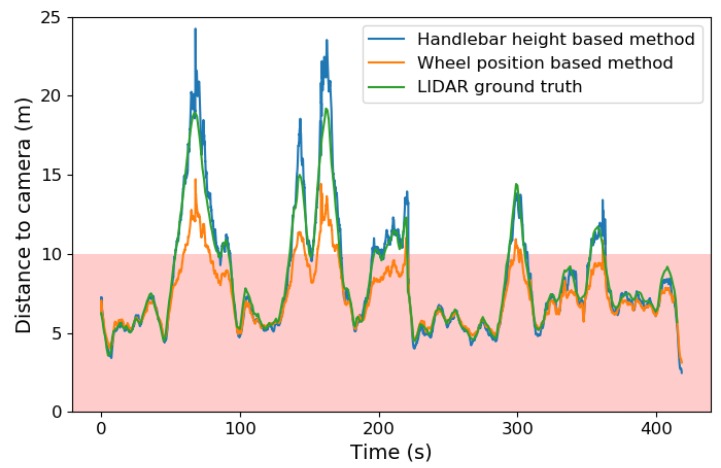
Measured distances on our dynamic test sequence. The draft zone is colored pink. The LiDAR data is considered ground truth.

To test the drafting detection algorithm in a realistic environment, a LiDAR system is used on a cargo bike to perform accurate distance measurements in a triathlon race-like situation. A flat road with no gradients or bends is considered in this test set-up. The LiDAR of the Velodyne VLP 16 type provides a 3D point cloud of the environment through which the measured distance of the drafting detection system can be compared with the LiDAR distance with an accuracy of 0.1 m. The arrangement is shown in Figure 11. The measurements from our distance estimation methods and the LiDAR ground truth from the sequence are shown in Figure 12.

**Figure 13:**
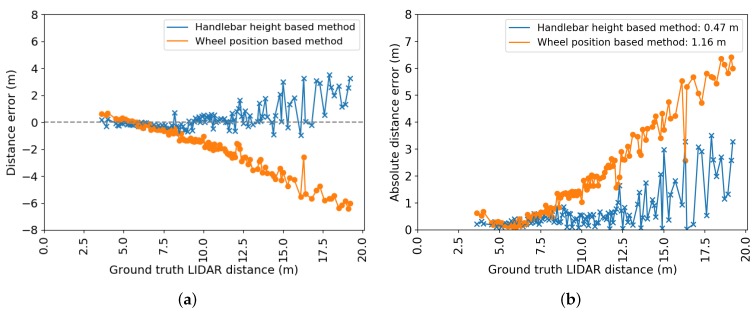
Error plots for the dynamic test for both distance estimation methods. (**a**) Signed error; (**b**) absolute error.

Figure 13 shows the signed and absolute distance errors calculated for 416 ground truth LiDAR distances for both the handlebar height based method and the WPm. The LiDAR ground truth distances vary from approximately 3 to 19 m. The absolute error graph demonstrates that the HHm is more accurate than the WPm, with average absolute errors of 0.47 and 1.16 m, respectively.

The signed error graphs also indicate that both distance estimation methods are prone to systematic, distance dependent errors. For the WPm, this bias is most likely caused by a significant camera tilt change between the measurement of this angle and the beginning of the test. The bias for the HHm indicates a downward tilt and/or $h_{2}>h_{1}$.

To further improve the overall distance measurement results in future work, the neural network of the detector could be retrained with a larger training set, notably with cyclists far from the camera bicycle. Other network architectures can also be considered, keeping in mind that the processing speed still needs to be adequate. However, the largest benefit could be obtained by avoiding the systematic errors as much as possible, as explained in Section 2.3.4.

### 3.2. Drafting Probability

#### 3.2.1. Estimation of Measurement Noise Level

The distance estimation accuracy depends on the accuracy of the parameters in formulae Equations (4) and (A3) and the approximations used to obtain Equation (6). We modeled the distributions of these parameter measurements as normal distributions, for set-ups similar to the one used in our dynamic test. The modeled mean and standard deviation of each parameter can be found in Table 2.

**Figure 14:**
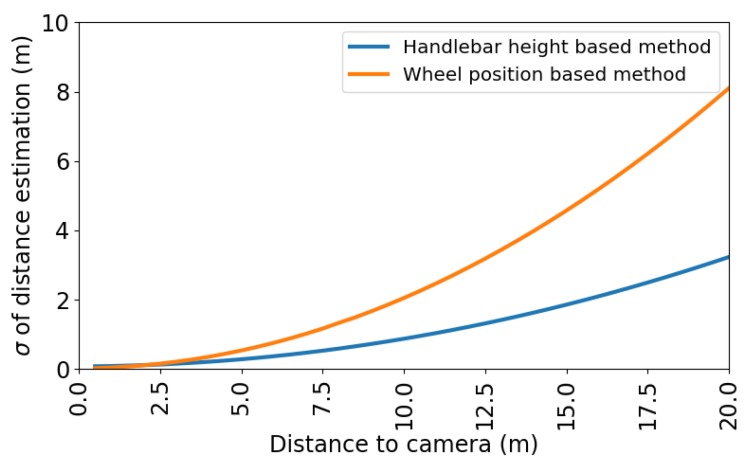
Simulated standard deviations for both distance estimation methods.

We assume all errors are uncorrelated. Thus, these errors propagate to the estimated distances as follows [24]:

(14) $$\begin{array}{cc} \sigma_{x_{w}}= & \sqrt{\frac{\partial x_{w}}{\partial h_{1}}\sigma_{h_{1}}^{2}+\frac{\partial x_{w}}{\partial f}\sigma_{f}^{2}+\frac{\partial x_{w}}{\partial y_{w}}\sigma_{y_{w}}^{2}+\frac{\partial x_{w}}{\partial \theta }\sigma_{\theta }^{2}}, \end{array}$$

(15) $$\begin{array}{cc} \sigma_{x_{h}}= & \sqrt{\frac{\partial x_{h}}{\partial h_{1}}\sigma_{h_{1}}^{2}+\frac{\partial x_{h}}{\partial h_{2}}\sigma_{h_{2}}^{2}+\frac{\partial x_{h}}{\partial f}\sigma_{f}^{2}+\frac{\partial x_{h}}{\partial y_{h}}\sigma_{y_{h}}^{2}+\frac{\partial x_{h}}{\partial \theta }\sigma_{\theta_{approx}}^{2}}\;, \end{array}$$

where $\sigma_{x_{w}}$ and $\sigma_{x_{h}}$ are the estimated standard deviations of the errors on the WPm and the HHm, respectively. These standard deviations are distance dependent and plotted in Figure 14.

Note that the HHm is again clearly more accurate than the WPm in the error simulations. This observation agrees with the sensitivity w.r.t. θ (Section 2.3.3) and our experiments in Section 3.1. With the obtained standard deviations, the drafting probability can be calculated according to the method explained in Section 2.4. We will further analyze the drafting probability with distance estimations obtained from the HHm.

#### 3.2.2. Drafting Violation Probability

**Figure 15:**
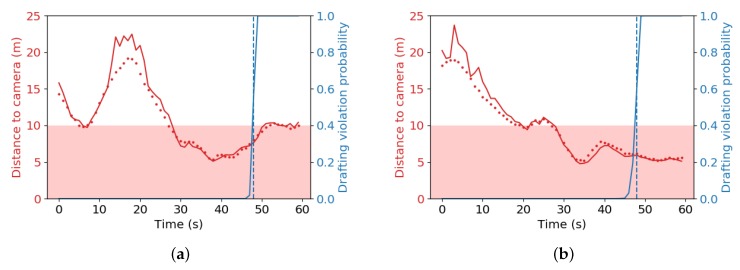
Drafting violation probability in two parts of our dynamic test. The distance to the cyclist was estimated with the HHm (red solid line). The ground truth distances are shown with red dots as markers. The draft zone is colored pink. The evolution of the drafting violation probability is superimposed on the same graphs (blue solid line). A blue dashed vertical line indicates when $P(v_{1:n}|x_{1:n})>0.5$ for the first time. The sampling period for both the LiDAR and the sampled distance measurements was 1s. (**a**) Cyclist behind driving to edge of draft zone, then further away, before approaching again and eventually entering the draft zone after approximately 27 s; (**b**) cyclist behind driving to edge of draft zone, stays close to edge, before eventually entering the draft zone after approximately 27 s.

We calculated the drafting violation probability for two subsequences of real data from our dynamic test, with video input similar to Figure 11a. We assume the probabilities are reset in between, such that multiple violations can be detected. The results are demonstrated in Figure 15.

For the dynamic test, the HHm notably overestimates the distance to the cyclist behind when the distance is large due to systematic errors. However, these errors barely influence the probabilities, since the measurement error for distances close to or below the drafting limit is much smaller. The drafting violation is detected rapidly and correctly in both subsequences.

We further analyzed the behavior of the drafting probability estimator by means of simulated distances with added measurement noise. In every simulation, the cyclist behind is set to start at the edge of the drafting zone, at a distance of 10 m. The cyclist then either moves inside of or away from the drafting zone at a constant relative velocity, such that he arrives at a new distance $x_{d}$ after 10 s. Then, the cyclist stays at this new position indefinitely, and the calculated drafting violation probability is calculated with Equation (13) with $\sigma_{x}=\sigma_{x_{t}}$ obtained as explained in Section 3.2.1.

Distances $x_{d}$ from 7 m to 11 m are considered, for sampling periods $T_{s}$ of 0.5 s, 1 s, 2 s and 4 s. The simulated measurement noise was derived from the distributions in Table 2. The simulated noisy values for $h_{1}$, $h_{2}$, *f* and θ (all mostly caused by initial measurements) were kept constant during 1 iteration of the experiment. Only the noise for $y_{h}$ was randomly resampled from the distribution for every measurement. 1000 iterations of each considered combination of $x_{d}$ and $T_{s}$ were performed.

**Figure 16:**
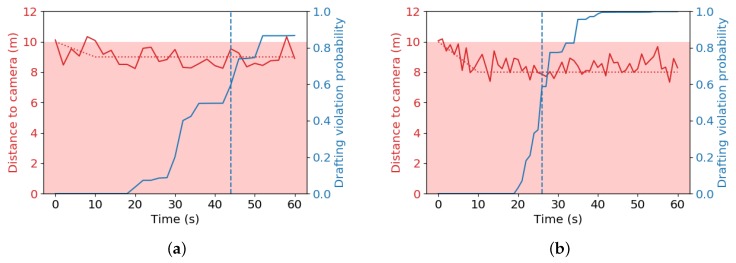
Example simulations of drafting violation probability over a given time frame. The cyclist enters the drafting zone in the beginning, and then settles at a new distance after 10 s (red dashed line). Gaussian noise is independently added to all parameters to simulate an estimated distance (red solid line). The draft zone is colored pink. The evolution of the drafting violation probability is superimposed on the same graphs (blue solid line). A blue dashed vertical line indicates when $P(v_{1:n}|x_{1:n})>0.5$ for the first time. (**a**) Cyclist riding at 9 m, sampling period 2 s, small systematic errors; (**b**) cyclist riding at 8 m, sampling period 1 s, systematic overestimation of the true distance because $h_{1}>h_{2}$.

**Figure 17:**
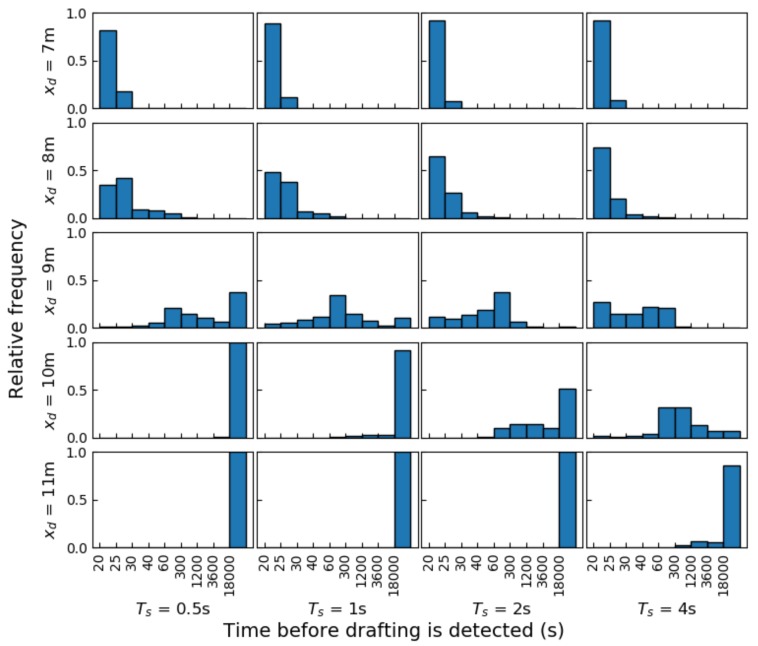
Histogram of time before $P(v_{1:n}|x_{1:n})>0.5$, with different sampling periods $T_{s}$ and for different distances $d_{f}$. One thousand iterations were performed for every combination of distance and sampling period. Ideally, the algorithm should award a penalty after $T_{L}=20$ s of drafting, and never when the cyclist stays out of the draft zone.

Note that 18,000 s=5 *h* is the typical time a participant spends on the cycling part of a full distance triathlon, so times longer than that are not investigated. Examples of these simulations are demonstrated in Figure 16. Each simulation yields a certain time until drafting is detected, whether this detection is desired or not. Distances below the drafting limit should be detected soon after the time limit. An overview with a histogram of the detection times from our simulations can be found in Figure 17.

The results demonstrate that, for distances above the drafting limit, a high sampling rate (low sampling period) is preferred, such that no drafting penalties are wrongfully awarded. Conversely, a lower sampling rate typically awards a drafting penalty faster and is thus the preferred option for distances below the drafting limit.

Theoretically, the resulting drafting violation probabilities should be similar when the underlying date is the same, regardless of the sampling rate. However, we made the assumption that all measurements are independent, which is typically not true when measuring the distance to the same object for an extended period of time. Furthermore, we assume implicitly that an athlete that is drafting at two successive samples, never exits the drafting zone in-between. This assumption might be too strict when the sampling period $T_{s}$ is large and can cause false positives. To avoid false positives, we recommend a sampling period of at most 2 s.

When riding just below the drafting limit (e.g., at 9 m), the drafting probability is clearly sensitive to potential distance estimation errors, and the time before drafting is detected is thus highly variable. The system would have less false negatives when $\sigma_{x}$ is small close to the drafting limit. To be fairly used in a race context, the rule enforcers need to take this into account. An alternative is to consider only awarding penalties for athletes riding at a distance significantly below the limit.

#### 3.2.3. Current Limitations and Future Work

The proposed approach still has a few limitations in its current form. We conclude this section with a brief discussion of these limitations and potential strategies to overcome them in future work.

1. In its current form, the proposed system is only able to track one cyclist behind at a time. A more complex track management system should be added such that multiple cyclists can potentially be followed throughout the sequence.
2. The values for $\sigma_{x_{w}}$ and $\sigma_{x_{h}}$ were now only estimated with the use of simulations. To more accurately estimate the parameters, real training data is required, with different cameras, bicycles, camera heights, tilt angles, as well as in different environments, with accurate ground truth, like we obtained in our dynamic test.
3. We have assumed all distance measurements are independent. However, in practice, the measured distance between two cyclists rarely varies significantly between successive frames. This can be observed by comparing the real data extracted from the dynamic test (Figure 15) with the simulations (Figure 16). Thus, low pass filtering or the incorporation of a temporal model (e.g., a Kalman filter) with transitional probabilities could further increase the accuracy of the measurement error model. An additional potential benefit of a Kalman filter is that it automatically estimates the measurement error, which can further optimize the estimation of the drafting violation probability.
4. As mentioned before and verified in this section, the estimated probability depends on the sampling rate. In addition, the exact moments the first sample is selected (just at the beginning of drafting or not) might influence the results, notably at low sampling rates and when the actual drafting time is close to the limit. The rule enforcers should be well aware of these characteristics and standardize the sampling settings and/or allow for enough buffer for questionable cases. This can, e.g., be realized by allowing slightly shorter distances and longer drafting times, or by imposing a drafting probability threshold that differs from 50%.

## 4. Conclusions

In this paper, a proof of concept for a drafting detection system in triathlon was proposed. The system is composed of four important building blocks: object detection, object tracking, distance determination, and drafting violation probability estimation. Detecting and then following the closest cyclist through the different scenes ensures a continuous monitoring over time. An average precision of 88.27% for detected bicycles was obtained on the test set after tracking. The Handlebar Height-Based method (HHm) method appears to be the most accurate one for distance determination, as the Wheel Position-Based method (WPm) is too sensitive to potential tilt angle changes or poor tilt angle estimation. In the static situation test, the average absolute error over all ground truth distances was 0.89 and 1.46 m for the HHm and the WPm, respectively. Furthermore, a dynamic test was conducted with LiDAR distances as the ground truth. Over all ground truth distances, this experiment shows that the HHm has an average absolute error of 0.47 m. For the WPm, this error is 1.16 m.

In addition, a drafting violation probability estimation was developed, which checks how likely it is that the drafting rule has been broken over a period of time. When using an appropriate sampling period of at most or 2 s, simulations demonstrate that the calculated probabilities are very useful to detect drafting at 8 m or below. The time before drafting is detected just below the drafting limit is highly variable, however. Nevertheless, with these settings, no unjustified penalties are awarded within a realistic time window. The proposed system shows promise to enter a triathlon and duathlon in order to obtain fairer races when further developed.

## Figures and Tables

**Table 1:** Approximate relative errors with respect to the different parameters in the distance estimation formulae (4)–(5) derived from sensitivity analysis. We assume that the tilt angle θ is small and ∂u/∂v≈Δu/Δv for all parameters *u* and *v*.

Method	WPM	HHm
**Relative errors**	$\frac{\Delta x_{w}}{x_{w}}$	$\frac{\Delta x_{h}}{x_{h}}$
Camera height $h_{1}$	$\frac{\Delta h_{1}}{h_{1}}$	$\frac{\Delta h_{1}}{h_{1}}$
Focal length *f*	$\frac{\Delta f}{f}$	$\frac{\Delta f}{f}$
Tilt angle θ	$-\Delta \theta \frac{2}{\sin\left(2\alpha \right)}$	−Δθtanα
Bottom position of bounding box $y_{w}$	$\frac{\Delta y_{w}}{y_{w}}$	not relevant
Height of bounding box $y_{h}$	not relevant	$-\frac{\Delta y_{h}}{y_{h}}$

**Table 2:** Estimated mean and standard deviations of the normally distributed parameters in our simulations. These estimations were obtained through observation of realistic measurements. Note that the standard deviation for $h_{2}$ is significantly bigger than the standard deviation for $h_{1}$ because the latter can be measured much easier in a real application. Furthermore, we assume that the standard deviation for the error on θ is much smaller when it is actually measured (e.g., in Equation (4)) than when we assume it is always 0 (e.g., in Equation (6)). Finally, we note that the means of $y_{w}$ and $y_{h}$ are both distance dependent, so we did not include them in this table.

Parameter	Mean	Error Standard Deviation σ (estimated)
Camera height $h_{1}\left(m\right)$	0.900	0.005
Bicycle height $h_{2}\left(m\right)$	0.900	0.025
Focal length f(pixels)	1000	25
Tilt angle $\theta_{approx}\left({}^{\circ}\right)$, with approximation Equation (6)	0	5
Tilt angle θ(°), without approximation	0	1
Bottom position of bounding box $y_{w}\left(\mathrm{pixels}\right)$	not relevant	5
Bounding box height $y_{h}\left(\mathrm{pixels}\right)$	not relevant	$5\sqrt{2}$

## Appendix A Distance for *h*_1_ ≠ *h*_2_

The distance from the camera to the bicycle behind can be written w.r.t. the position of the bottom of the detected bounding box as

(A1) $$x_{w}=\frac{h_{1}\left(f+y_{w}\tan\theta \right)}{-y_{w}+f\tan\theta }\;,$$

as demonstrated in Equation (4). $y_{w}$ can be rewritten as a function of the top of the bounding box and its height $y_{h}$ as

(A2) $$y_{w}=f\frac{\tan\theta +\frac{h_{2}-h_{1}}{x_{h}}}{1-\frac{\left(h_{2}-h_{1}\right)\tan\theta }{x_{h}}}-y_{h}\;.$$

Substituting $y_{h}$ with the expression in Equation (A2) into Equation (A1) yields

(A3) $$\begin{array}{c} \frac{y_{h}{x_{h}}^{2}+\left(y_{h}\left(2h_{1}-h_{2}\right)\tan\theta -fh_{2}\left(1-\tan^{2}\theta \right)\right)x_{h}+y_{h}h_{1}\left(-h_{2}+h_{1}\right)\tan^{2}\theta }{\left(-h_{2}+h_{1}\right)\tan\theta +x_{h}}=0\;. \end{array}$$

This equation yields two solutions when solved for $x_{h}\neq \left(h_{1}-h_{2}\right)\tan\theta$. However, one solution always yields an unrealistically small, zero or negative estimated distance and can easily be discarded. Note that the product of both solutions is

(A4) $$\begin{array}{cc} x_{h,1}x_{h,2}= & \frac{y_{h}h_{1}\left(-h_{2}+h_{1}\right)\tan^{2}\theta }{y_{h}} \end{array}$$

(A5) $$\begin{array}{cc} = & h_{1}\left(-h_{2}+h_{1}\right)\tan^{2}\theta \;. \end{array}$$

Hence, the following observations can be made:

- When either $h_{1}=h_{2}$ or θ=0, there is always one zero solution.
- When $h_{1}>h_{2}$, there is exactly one negative solution.
- When $h_{2}>h_{1}$, both solutions have the same sign, but one solution will have a much smaller value than the other, e.g., let $h_{2}=0.8$ m, $h_{1}=0.85$ m, and θ=10°. From Equation (A5), an object at (real distance) 10 m can theoretically be confused with an object at 132 μm, which in practice cannot even be fully captured on the image plane.

Thus, when only considering objects in front of the camera which are fully visible, the largest value of $x_{h}$ can generally be used as the estimated distance.

## Appendix B Sensitivity Analysis of Distance Formulae

To perform sensitivity analysis of the distance estimation formulae from Section 2.3, we partially derive the expressions Equations (4) and (5) to the most relevant parameters. To simplify the resulting expressions, we generally assume the tilt angle θ is small and $h_{1}\approx h_{2}$. Furthermore, $x_{w}=x_{h}=x$.

### Appendix B.1 Height of the Camera h_1_

(A6) $$\begin{array}{cc} \frac{\partial x_{w}}{\partial h_{1}} & =\frac{f+y_{w}\tan\theta }{f\tan\theta -y_{w}}=\frac{x_{w}}{h_{1}} \end{array}$$

(A7) $$\begin{array}{cc} \frac{\partial x_{h}}{\partial h_{1}} & =\frac{f\left(1+\tan^{2}\theta \right)-y_{h}\tan\theta }{y_{h}}=\frac{x_{h}}{h_{1}} \end{array}$$

### Appendix B.2 Focal Length of the Camera f

(A8) $$\begin{array}{cc} \frac{\partial x_{w}}{\partial f} & =-\frac{h_{1}y_{w}\left(1+\tan^{2}\theta \right)}{{\left(f\tan\theta -y_{w}\right)}^{2}}=-\frac{x_{w}^{2}y_{w}\left(1+\tan^{2}\theta \right)}{h_{1}{\left(f+y_{w}\tan\theta \right)}^{2}} \end{array}$$

(A9) $$\begin{array}{cc} \; & \Rightarrow {\left.\frac{\partial x_{w}}{\partial f}\right|}_{\theta =0}=-\frac{x_{w}^{2}\left(-y_{h}\right)}{h_{1}f^{2}}\overset{\left(6\right)}{\approx }\frac{x_{w}}{f} \end{array}$$

(A10) $$\begin{array}{cc} \frac{\partial x_{h}}{\partial f} & =\frac{h_{1}\left(1+\tan^{2}\theta \right)}{y_{h}} \end{array}$$

(A11) $$\begin{array}{cc} \; & \Rightarrow {\left.\frac{\partial x_{h}}{\partial f}\right|}_{\theta =0}=\frac{h_{1}}{y_{h}}\overset{\left(6\right)}{\approx }\frac{x_{h}}{f} \end{array}$$

### Appendix B.3 Tilt Angle θ

(A12) $$\begin{array}{cc} \frac{\partial x_{w}}{\partial \theta } & =-\frac{h_{1}\left(f^{2}+y_{w}^{2}\right)\left(\tan^{2}\theta +1\right)}{{\left(f\tan\theta -y_{w}\right)}^{2}} \end{array}$$

(A13) $$\begin{array}{cc} \; & \Rightarrow {\left.\frac{\partial x_{w}}{\partial \theta }\right|}_{\theta =0}=-\frac{h_{1}\left(f^{2}+y_{w}^{2}\right)}{y_{w}^{2}}\overset{\left(6\right)}{\approx }-\frac{y_{h}}{f}\frac{f^{2}+y_{w}^{2}}{y_{w}^{2}}x_{w}\approx -x_{w}\frac{2}{\sin\left(2\alpha \right)} \end{array}$$

(A14) $$\begin{array}{cc} \frac{\partial x_{h}}{\partial \theta } & =\frac{h_{1}\left(\tan^{2}\theta +1\right)\left(2f\tan\theta -y_{h}\right)}{y_{h}} \end{array}$$

(A15) $$\begin{array}{cc} \; & \Rightarrow {\left.\frac{\partial x_{h}}{\partial \theta }\right|}_{\theta =0}=-h_{1}\overset{\left(6\right)}{\approx }-\frac{y_{h}}{f}x_{h}\approx -x_{h}\tan\alpha \end{array}$$

### Appendix B.4 Bottom Position of Detected Bounding y_w_

(A16) $$\begin{array}{cc} \frac{\partial x_{w}}{\partial y_{w}} & =\frac{h_{1}f\left(\tan^{2}\theta +1\right)}{{\left(f\tan\theta -y_{w}\right)}^{2}}=\frac{x_{w}^{2}f\left(\tan^{2}\theta +1\right)}{h_{1}{\left(f+y_{w}\tan\theta \right)}^{2}} \end{array}$$

(A17) $$\begin{array}{cc} \; & \Rightarrow {\left.\frac{\partial x_{w}}{\partial y_{w}}\right|}_{\theta =0}=\frac{x_{w}^{2}}{h_{1}f}\overset{\left(6\right)}{\approx }\frac{x_{w}}{y_{h}} \end{array}$$

### Appendix B.5 Height of Detected Bounding Box y_h_

(A18) $$\begin{array}{cc} \frac{\partial x_{h}}{\partial y_{h}} & =-\frac{fh_{1}\left(\tan^{2}\theta +1\right)}{y_{h}^{2}} \end{array}$$

(A19) $$\begin{array}{cc} \; & \Rightarrow {\left.\frac{\partial x_{h}}{\partial y_{h}}\right|}_{\theta =0}=-\frac{fh_{1}}{y_{h}^{2}}\overset{\left(6\right)}{\approx }-\frac{x_{h}}{y_{h}} \end{array}$$
